# Spatially defined transcriptomic changes across the hippocampus of J20 mice

**DOI:** 10.1002/alz.092346

**Published:** 2025-01-03

**Authors:** Millie Sander, Katie Lunnon, Clive G Ballard, Jonathan T Brown

**Affiliations:** ^1^ University of Exeter, Exeter United Kingdom; ^2^ University of Exeter, Exeter, Devon United Kingdom

## Abstract

**Background:**

The J20 mouse is an established model of amyloid pathology, exhibiting neuropathological and behavioural symptoms reflective of human Alzheimer’s disease (AD). Previous work, conducted by Castanho et al (2020), revealed transcriptomic change in the hippocampus of J20 mice to be associated with the accumulation of amyloid pathology. Here, we investigated the spatial distribution of such transcriptomic changes using novel spatial transcriptomic technology.

**Method:**

10µm fresh‐frozen brain slices were collected from the brains of J20 mice and WT controls. Slices were mounted on 10x genomics visium slides and processed according to the manufacturer recommendations. A total of 47 spatial clusters were identified, 6 of which corresponded to the hippocampus. Differential expression analysis was conducted on the hippocampus and sub‐regions of interest (CA1 & CA3 pyramidal cell layers, and dentate gyrus granule cell layer). High dimensional weighted gene co‐expression network analysis (hdWGCNA) was conducted to identify spatially‐resolves networks of co‐expressed genes.

**Result:**

Various differentially expressed genes (DEGs) were identified across the hippocampus of J20 mice compared with WT controls. Many of said DEGs were found to be cluster‐specific, indicating transcriptomic change to be specific to hippocampal sub‐regions. hdWGCNA modules were also highly region‐specific. Many of said genes/hdWGCNA modules, and the associated biological pathways, were associated with AD pathology and behaviour.

**Conclusion:**

Overall, these results demonstrate region‐specific transcriptional changes in the hippocampus of J20 mice compared with WT controls. DEGs and networks of co‐expressed genes were found to be associated with disease‐associated pathways, giving insight into region‐specific pathological changes with disease.

